# Multimodality infarct identification for optimal image-guided intramyocardial cell injections

**DOI:** 10.1007/s12471-014-0604-2

**Published:** 2014-10-21

**Authors:** F. J. van Slochteren, R. van Es, S. Koudstaal, T. I. G. van der Spoel, J. P. G. Sluijter, J. Verbree, R. H. R. Pruim, J. P. W. Pluim, T. Leiner, P. A. Doevendans, S. A. J. Chamuleau

**Affiliations:** 1 Department of Cardiology, University Medical Center Utrecht, E03.511, PO Box 85500, 3508 GA Utrecht, the Netherlands; 8 Department of Radiology, University Medical Center Utrecht, E03.511, PO Box 85500, 3508 GA Utrecht, the Netherlands; 2 Department of Technical Medicine, University of Twente, Enschede, the Netherlands; 3 Imaging Sciences Institute, University Medical Center Utrecht, QS.459, Heidelberglaan 100, 3584 CX Utrecht, the Netherlands; 4 Department of Radiology, University Medical Center Utrecht, E01.102, Heidelberglaan 100, 3584 CX Utrecht, the Netherlands; 5 Interuniversity Cardiology Institute of the Netherlands (ICIN), Utrecht, the Netherlands; 6 Department of Radiology, Leiden University Medical Center, the Netherlands, Albinusdreef 2, 2333 ZA Leiden, the Netherlands; 7 Donders Institute, Radboud University Nijmegen, the Netherlands, Geert Grooteplein-Noord 21, 6525 EZ Nijmegen, the Netherlands

**Keywords:** Cardiac regenerative therapy, Intramyocardial injections, Electromechanical mapping, Myocardial fibrosis, MRI, Late Gadolinium enhancement

## Abstract

**Background:**

Intramyocardial cell injections in the context of cardiac regenerative therapy can currently be performed using electromechanical mapping (EMM) provided by the NOGA®XP catheter injection system. The gold standard technique to determine infarct size and location, however, is late gadolinium enhanced magnetic resonance imaging (LGE-MRI). In this article we describe a practical and accurate technique to co-register LGE-MRI and NOGA®XP datasets during the injection procedures to ultimately perform image-guided injections to the border zone of the infarct determined by LGE-MRI.

**Materials and methods:**

LGE-MRI and EMM were obtained in three pigs with chronic myocardial infarction. MRI and EMM datasets were registered using the in-house developed 3D CartBox image registration toolbox consisting of three steps: 1) landmark registration, 2) surface registration, and 3) manual optimization. The apex and the coronary ostia were used as landmarks.

**Results:**

Image registration was successful in all datasets, and resulted in a mean registration error of 3.22 ± 1.86 mm between the MRI surface mesh and EMM points. Visual assessment revealed that the locations and the transmural extent of the infarctions measured by LGE-MRI only partly overlap with the infarct areas identified by the EMM parameters.

**Conclusions:**

The 3D CartBox image registration toolbox enables registration of EMM on pre-procedurally acquired MRI during the catheter injection procedure. This allows the operator to perform real-time image-guided cell injections into the border zone of the infarct as assessed by LGE-MRI. The 3D CartBox thereby enables, for the first time, standardisation of the injection location for cardiac regenerative therapy.

**Electronic supplementary material:**

The online version of this article (doi:10.1007/s12471-014-0604-2) contains supplementary material, which is available to authorized users.

## Introduction

Previous studies have shown that injection of stem/progenitor cells into the border zone of the infarcted area in the context of cardiac regenerative therapy helped to stimulate cardiac repair and protection via cell-to-cell contact and secretion of paracrine factors [1–7]. However, therapeutic effects may rely on the delivery and retention of the regenerative therapeutics on a location where oxygen and nutrients are available to enable survival [8]. Hence, accurate identification of viable tissue in the proximity of the infarct is of great importance. Via intramyocardial injection catheters stem/progenitor cells or biomaterials can be injected in a minimally invasive fashion into the myocardium. Injection locations can be chosen based on tissue viability measures obtained from electromechanical mapping (EMM), or a-priori knowledge about the infarct location [9]. The NOGA®XP intramyocardial injection system [10] provides a three-dimensional (3D) magnetic tracking technology and allows the assessment of local electrical and mechanical tissue characteristics. Local unipolar and bipolar depolarisation potentials and relative catheter tip displacements (linear local shortening) are measured at multiple locations on the left ventricular endocardium. These measurements are interpolated to obtain a three-dimensional reconstruction which is used to guide cell injections. This technique is currently used in clinical practice [11–16]. Measurements can however not, be performed in regions that are susceptible for arrhythmias and measurements are interpolated in regions where no measurements are taken. Furthermore, cut-off values of EMM parameters to identify areas with different viability/perfusion/transmurality vary greatly between studies [15]. Altogether this approach is not reproducible and prone to errors regarding accurate and detailed identification of the infarct border zone. Since the non-transmural border zone of the infarction is believed to be the preferred delivery site of the stem cell therapeutics [1], it is crucial for it to be optimally defined during the injection procedure. We hypothesise that combining the gold standard measure of infarct size and location by late gadolinium enhanced magnetic resonance imaging (LGE-MRI) or other MRI techniques [17] and practical guidance (NOGA®XP) would further optimize the cell delivery location, and lead to a uniform injection strategy. This approach enables accurate selection and targeting of the infarct border zone with a distinct infarct transmurality with a value between 0 and 100 %. In this study we describe the development of a practical software toolbox (3D CartBox) that enables real-time image-guided cell injections. The 3D CartBox registers NOGA®XP catheter positions on pre-procedurally acquired MRI images to perform intramyocardial injections to locations with an a priori identified distinct infarct transmurality. In addition, the 3D CartBox can be used to further specify the definition of border zone of the infarct [8].

## Materials and methods

**Fig. 1:**
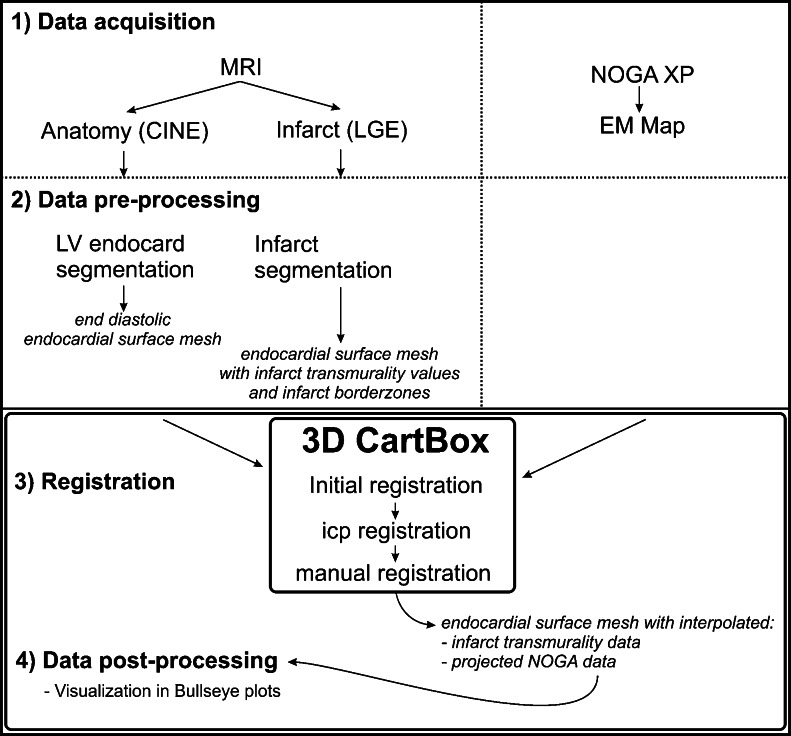
Workflow of the image processing steps that are necessary to use the 3D CartBox toolbox

**Fig. 2:**
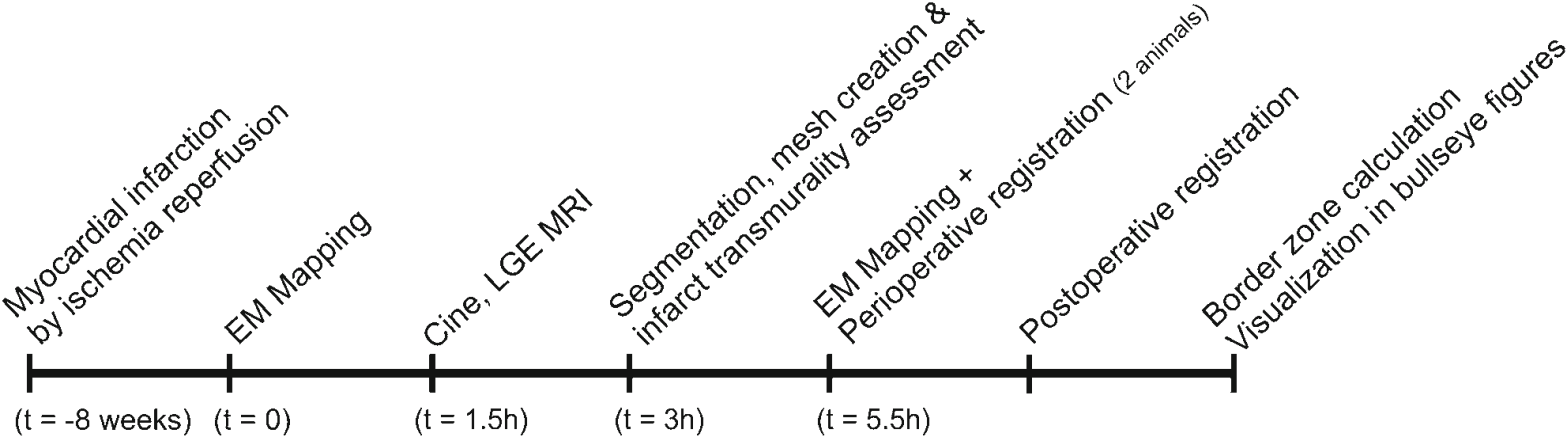
Time line of the experiments. The second mapping procedure was performed in two pigs

The 3D CartBox is a Matlab-based software toolbox to register NOGA®XP catheter positions on an endocardial surface mesh derived from MRI. Prior to registration two image processing steps are necessary: 1) Data acquisition, and 2) Data pre-processing as illustrated in Fig. 1. The 3D CartBox is used for the registration and consists of three phases: 1) initial registration, 2) iterative closest point (ICP) registration, and 3) manual registration. After the registration process post-processing is performed to visualise the data in bullseye plots. In the development process described in this study the 3D CartBox toolbox was applied to data of three pigs with a chronic myocardial infarction. The study design is illustrated in Fig. 2. The algorithms used in the 3D CartBox and in the pre- and post-processing steps are explained in detail in the supplementary data.

### Animals

All experiments were performed in accordance with the ‘Guide for the Care and Use of Laboratory Pigs’ prepared by the Institute of Laboratory Animal Resources and with prior approval by the Animal Experimentation Committee of the Faculty of Medicine, Utrecht University, the Netherlands. Three 6-month-old female Dalland Landrace pigs (60–70 kg; Central Institute for Animal Disease Control (IDDLO), Lelystad, the Netherlands) were pre-treated with clopidogrel 75 mg/day for 3 days and amiodarone 400 mg/day for 10 days. In all pigs myocardial infarction (MI) was induced by 75 min of percutaneous balloon occlusion of the proximal left circumflex coronary artery as previously described [18]. Eight weeks after MI the experiments were performed as shown in Fig. 2.

### Data acquisition

The in vivo MRI images were acquired using a 1.5 T Philips Medical Systems Achieva scanner. The MRI sequences for the cine and the LGE MRI acquisitions are described in the supplementary data. The NOGA® XP system (Biosense Webster, Cordis, Johnson & Johnson, USA) version 1.1.43 was used equipped with a 7 French NOGA mapping catheter (Biosense Webster, Cordis, Johnson & Johnson, Diamond Bar, USA) for the mapping. The left ventricle was entered via the left carotid artery, and retrograde passage through the aortic valve. Readout of the catheter tip location was done at end-diastole using R-wave triggering, thereby providing only end-diastolic tip locations for registration. Electrocardiograms were filtered at 30–400 Hz (bipolar) and 1–240 Hz (unipolar). The EMM datasets were acquired in consideration of the criteria for good electromechanical mapping [15]. Points acquired in the left and right coronary ostia and the apex served as anatomical landmarks and are used for the first registration step. The apex location was taken as the most outward point that was reached in the apical region confirmed by fluoroscopy. To obtain data from the NOGA®XP system in a real-time fashion, the system was modified to enable read-only access from an external computer running the 3D CartBox.

### Data pre-processing

Data pre-processing consists of 1) segmentation of the left ventricle using the cine images [23], 2) segmentation of the infarct using the LGE images [24], 3) calculation of the infarct transmurality, 4) projection of the infarct transmurality on the endocardial surface mesh derived from the end-diastolic cine images, and 5) calculation of the infarct transmurality border zone, the preferred site for cell injections. The details of the pre-processing steps are described in the supplementary data.

### Registration

During initial registration the raw NOGA®XP dataset and the MRI datasets are registered coarsely based on the anatomical landmarks using a closed-form least squares approach [19, 20]. The anatomical landmarks used are the left and the right coronary ostia and the apex as previously described [21]. After acquiring points in all regions of the left ventricular endocardium, an ICP algorithm [22] was applied to optimise the registration. If necessary the registration was manually optimised by adjusting the registration interactively. The algorithms used for the registration are explained in detail in the supplementary data. The accuracy of the registration was expressed by the registration error being the mean ± standard deviation of the shortest distance from each EMM point to the cine mesh surface as previously described [21]. The relevance of the registration error measure used is pointed out in the supplementary data. To prevent interference by EMM points that were not located in the cine mesh (e.g. left ventricular outflow tract), these points were excluded for ICP registration, error calculation, and further processing.

### Perioperative image guidance

**Fig. 3:**
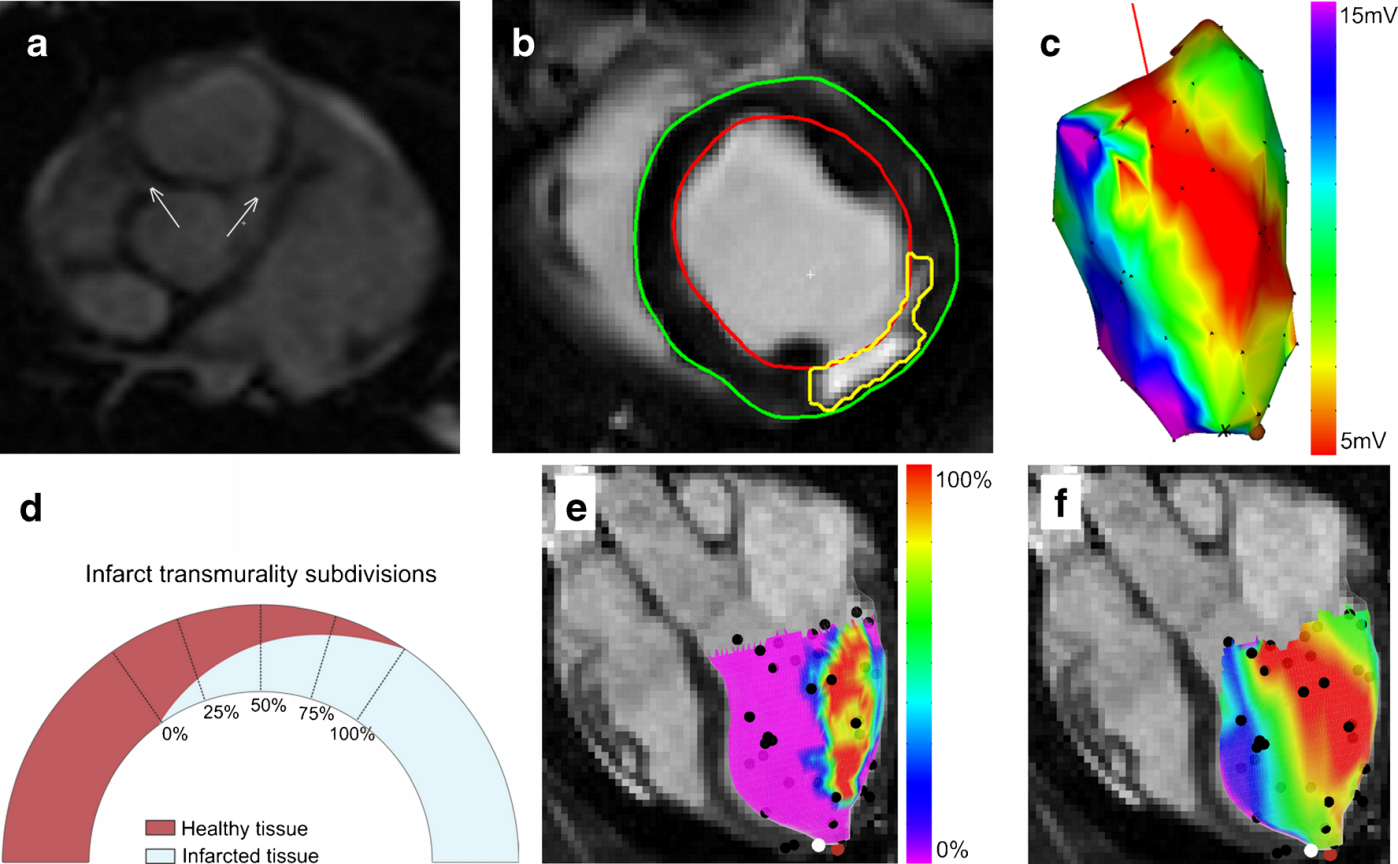
Short-axis balanced fast field echo image with coronary ostia (**a**). Segmentation of the epicardium (*green*), endocardium (*red*), scar area (*yellow*) on the short-axis LGE scans (**b**). NOGA®XP representation of unipolar voltage map (**c**). Subdivision of infarct transmurality (**d**). Infarct transmurality superimposed on cine mesh. Unipolar voltage projection superimposed on cine mesh (**f**). Coloured dots are: NOGA measurement points (*black*), the apex (*white*), annotated location (*brown*)

In this study perioperative registration was performed in two animals. After initial registration the acquired EMM points were visualised in the freely rotatable 3D endocardial surface mesh with projected infarct transmurality data. In addition the MR images are displayed as shown in Fig. 3e and f. Newly acquired NOGA®XP measurement points appeared on the screen. Free rotation of the mesh and the MR images during the image-guided procedure assures correct positioning of the needle before cell injection.

### Post-processing

For visualisation purposes both the infarct transmurality values and the infarct border zones, as well as the EMM data, were projected on the 3D endocardial surface mesh and bullseye plots using customised software.

### Statistical analysis

All data are presented as mean ± standard deviation (SD). A one-way ANOVA test was used to assess the difference between the number of points/cm^2^ in the different infarct transmurality areas. *P* < 0.05 was considered significant.

## Results

### Data pre-processing

MRI findings are listed in Table 1. The mean left ventricular ejection fraction (LVEF) was 42 ± 1 %. All LGE datasets showed a clear fibrotic area as shown in Fig. 3b. The LVEF shows that 8 weeks after myocardial infarction the pigs had mild heart failure, and comparable infarct sizes (27.5 ± 3.5 % of total left ventricular area). The 75–100 % infarct transmurality area was the smallest, and was absent in one pig. Infarct transmurality was assessed and subdivided in areas with 0, 0–25, 25–50, 50–75, and 75–100 % transmurality as illustrated in Fig. 3d. The mean endocardial surface areas in these subdivisions were: 73.6 ± 19.7, 7.2 ± 1.0, 9.5 ± 2.6, 5.8 ± 2.8, and 4.6 ± 5.2 cm^2^, respectively, thereby covering 72.5 ± 5.1, 7.2 ± 0.5, 9.6 ± 2.9, 6.4 ± 4.4, and 4.3 ± 4.3 % of the total LV endocardial surface area. The locations of the coronary ostia could be identified on the cine images (Fig. 3a). In all three pigs we were able to acquire EMM points in, or in the vicinity of, the coronary ostia. The mean number of EMM points was 66.3 ± 14.2.

**Table 1:** Results of cine and late enhancement magnetic resonance imaging of three animals

LV end-diastolic volume (ml)	128 ± 19
LV end-systolic volume (ml)	75 ± 11
LV ejection fraction (%)	42 ± 1
Myocardium volume (ml)	118 ± 18
Infarct volume (ml)	17 ± 3
LV area (cm^2^)	101 ± 21
Infarct area 0 % transmurality (cm^2^)	73.6 ± 19.7 (72.5 ± 5.1 %)
Infarct area 0–25 % transmurality (cm^2^)	7.2 ± 1.0 (7.2 ± 0.5 %)
Infarct area 25–50 % transmurality (cm^2^)	9.5 ± 2.6 (9.6 ± 2.9 %)
Infarct area 50–75 % transmurality (cm^2^)	5.8 ± 2.8 (6.4 ± 4.4 %)
Infarct area 75–100 % transmurality (cm^2^)	4.6 ± 5.2 (4.3 ± 4.3 %)

Data are expressed as mean ± SD

### Registration

The registration results are listed in Table 2. Perioperative registration was performed in two pigs to test the real-time use of the 3D CartBox toolbox (Fig. 2). During the real-time EMM procedures the cine mesh was successfully merged with the EMM based upon the anatomical landmarks and ICP registration. After application of ICP the mean surface registration error was 3.27 ± 1.93 mm. No manual optimisation of the registration was applied to the datasets. After exclusion of points located outside the cine mesh the mean registration error was 3.22 ± 1.86 mm. A cine mesh with projected infarct transmurality and unipolar voltage are shown in Fig. 3e and f. Figure 3c shows the corresponding NOGA®XP EMM. The average coverage of EMM points in the different infarct transmurality areas was 0.76 ± 0.44 points/cm^2^. There was no significant difference between the number of points/cm^2^ in each infarct transmurality area of all the different animals (*p* = 0.96).

**Table 2:** Three-dimensional electromechanical mapping and image registration results of three animals

Electromechanical mapping points			
Total number of points	192		
Points per animal	66.3 ± 14.2		
Distance EMM points to MRI mesh surface (mm)			
All EMM points	3.27 ± 1.93 mm based on 192 points		
All EMM points in cine mesh	3.22 ± 1.86 mm based on 183 points		
Points per infarct transmurality area			
0 %	53.3 ± 11.7	0.8 ± 0.3 points/cm^2^	
0–25 %	6.0 ± 3.6	0.8 ± 0.5 points/cm^2^	
25–50 %	6.0 ± 3.0	0.7 ± 0.4 points/cm^2^	ns (*p* = 0.96)
50–75 %	2.6 ± 1.5	0.4 ± 0.1 points/cm^2^	
75–100 %	4.5 ± 4.1	0.9 ± 1.2 points/cm^2^	

Data are expressed as mean ± SD

### Post processing

**Fig. 4:**
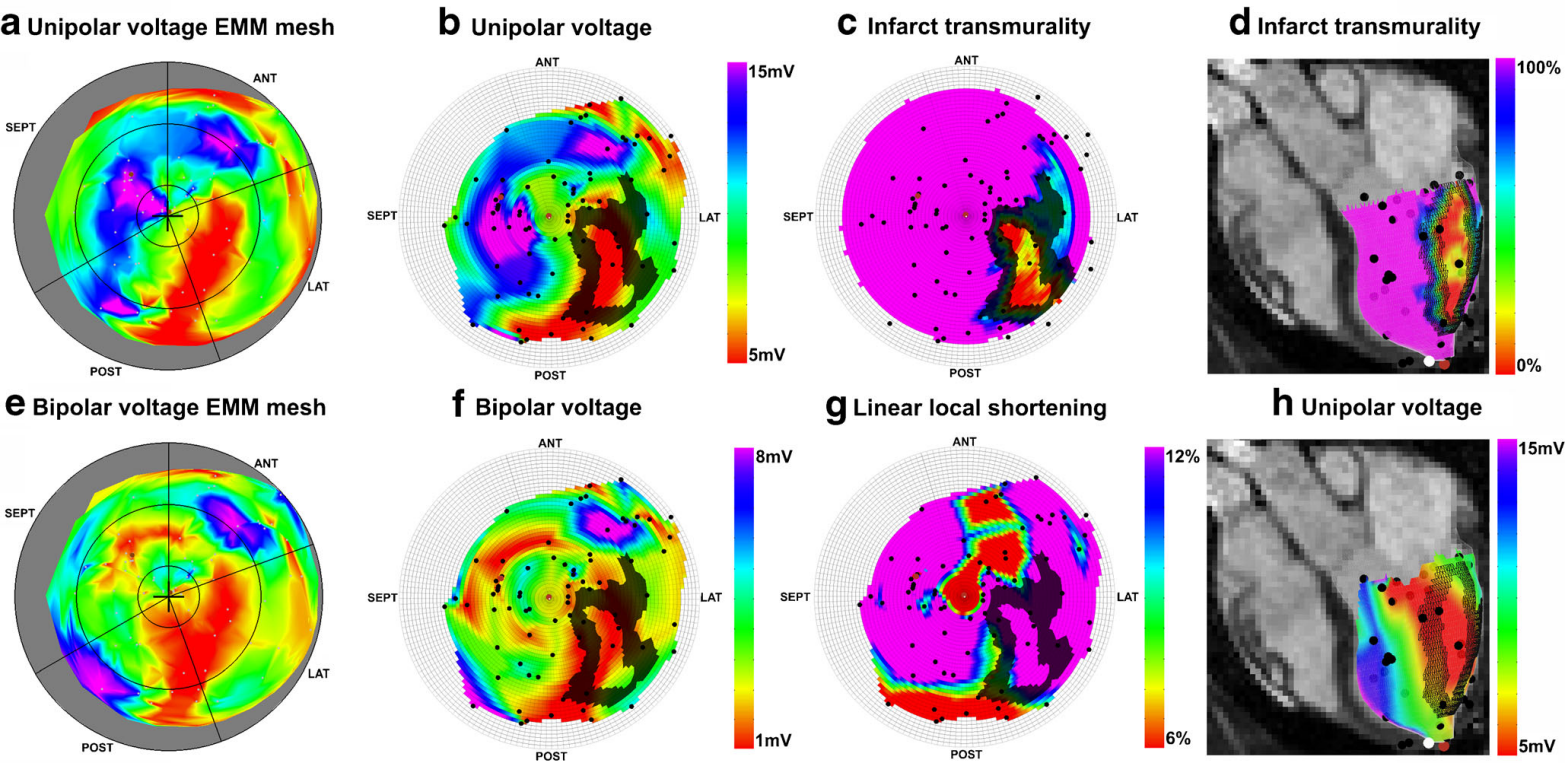
Typical bullseye plots of the NOGA system: unipolar voltage (**a**), bipolar voltage (**e**). And data projected on the cine mesh: unipolar voltage (**b**), bipolar voltage (**f**), infarct transmurality (**c**), linear local shortening (**g**). Infarct transmurality and unipolar voltages projected on the endocardial surface mesh placed in an end-diastolic long-axis cine MRI image (**d** and **h**). D and H represent the operator view during the image-guided injection procedure. Coloured dots are: NOGA measurement points (*black*), the apex (*white*), annotated location (*brown*). The *black* overlay in images **b**, **c**, **d**, **f**, **g**, **h** is a projection of the 50 % infarct transmurality border zone

To evaluate the projection of the EMM data on the endocardial surface mesh Fig. 4 shows bullseye figures of the unipolar and bipolar voltage as represented by the NOGA®XP system (a, e) and after projection on the cine mesh (b, f). Figure 4g represents the projection of LSS. From the comparison with the infarct transmurality depicted in Fig. 4c it can be observed that there is limited agreement between the EMM parameters and infarct transmurality. The 50 % infarct transmurality border zone determined by LGE-MRI is projected on the bullseye figures of the infarct transmurality (c), unipolar voltage (b), bipolar voltage (f), and linear local shortening (g). From these images it can be observed that the border zones of low unipolar and bipolar values show alignment with the 50 % infarct transmurality border zone. Figure 4d and h show the projection of the 50 % infarct transmurality border zone on the endocardial surface mesh as it is visualised during image-guided cell injection procedures.

## Discussion

In this study, the feasibility of perioperative integration of electromechanical data acquired with the NOGA® XP system with MRI data was shown for the first time. The in-house developed 3D CartBox image registration toolbox has allowed us to register the EMM dataset on a surface mesh derived from end-diastolic cine MRI images in real time with a mean surface registration error of 3.27 ± 1.93 mm. The three main findings of this study were: 1) Real-time use of the toolbox was feasible; 2) Visually a discrepancy was found between infarct assessment by EMM and LGE-MRI; 3) The 3D CartBox toolbox can be of additional value for infarct assessment and catheter guidance during cardiac cell injection procedures.

### Data acquisition and pre-processing

The LVEF in Table 1 showed that 8 weeks after myocardial infarction the pigs had mild heart failure, and comparable infarct sizes. The absence of a transmural infarction in one pig is most likely induced by the different response to the ischaemia reperfusion procedure due to biological variation. The number of points in the EMM (66.3 ± 14.2) just fulfilled the criteria for good electromechanical mapping as published [15]. During the mapping procedure the EMM points were acquired homogeneously spread over the endocardium. This was confirmed by the equal number of points in each area with a distinct infarct transmurality presented in Table 2, and can be observed from the distribution of EMM points in Fig. 4.

### Registration

Although acquiring more EMM points might have been beneficial for the registration, the homogeneous distribution of EMM points over the endocardium has secured a correct registration. Due to the longer acquisition time of the LGE images, gated LGE scans did not exactly represent the end-diastolic phase. To assure use of the end-diastolic ventricular shape for registration we chose to use the end-diastolic frame of the cine images for mesh creation, registration and projection of the EMM data. The exclusion of EMM points that were not located in the cine mesh was done to prevent registration based on EMM points that were located in areas of the left ventricle that were not visualised with MRI. Of the three cases in this study totally 9 mapping points were discarded for this reason. Consequently the registration using all EMM points has a slightly higher registration error: 3.27 ± 1.93 mm compared with 3.22 ± 1.86 mm for the map without the excluded points. Although this difference does not affect registration, it does prevent erroneous projection of EMM parameters on the cine mesh. The 10 times higher weighing of the apex during landmark registration and restriction of the ICP algorithm to 10° in the sagittal plane, and 20° in the transverse and coronal planes, were chosen empirically. The resulting surface registration error of 3.22 ± 1.86 mm was less than the value reported in research where CARTO merge was used [21]. The remaining error can be caused by respiratory-induced motion of the heart during the EMM procedure. The NOGA®XP system does not compensate for this.

### Post-processing

A high similarity is observed between the EMM maps produced by the NOGA®XP system and the EMM data projected on the cine mesh. The projection of the EMM data on the endocardial surface mesh via linear interpolation results in an accurate projection of the true endocardial surface measurements (Fig. 4a–b and e–f). From visual assessment it can be inferred that agreement between the infarct identification by EMM (Fig. 4b, f, e) and LGE-MRI (Fig. 4c) is the best with unipolar voltage, and poor with both bipolar voltage and linear local shortening. Although it cannot be ruled out completely, the small registration error makes it unlikely that this was the main origin of the poor agreement. Another explanation might be the occurrence of endocardial conducting tissue in regions with an infarct, mimicking normal tissue, or extension of the low voltage regions to neighbouring areas of non-transmural infarction through interpolation. Acquiring more points during the mapping procedure in the border zone may solve this problem, provided that this area is known, and not susceptible to arrhythmias due to catheter manipulation. Based on the rationale that 100 % transmurally infarcted myocardium is not a location where the stem cells are supplied with sufficient oxygen and nutrients [1], and in regions with 0 % transmurality there is no use for stem cells, we have chosen to show the 50 % infarct transmurality border zone on the bullseye figures in Fig. 4b, c, d, f, g, h. Visual assessment shows that the EMM parameters only partly identify the 50 % infarct transmurality border zone assessed by the gold standard LGE-MRI infarct assessment technique. The 3D CartBox toolbox enables accurate targeting of infarct border zones with a distinct infarct transmurality at any percentage between 0 and 100 % transmurality determined by LGE-MRI. Thereby the 3D CartBox, for the first time, enables objective specification of the target locations for intramyocardial injections in the context of cardiac regenerative therapy.

## Clinical implications

Incorrect injections of stem cells into the myocardium might jeopardize the success of cardiac regenerative therapy [8]. The combination of the gold standard fibrosis imaging technique for infarct and infarct border zone identification, and a catheter navigation technique to guide injections, is a crucial step to target the infarct border zone more accurately. This approach could lead to shorter injection procedures, less necessity for the use of fluoroscopy to confirm the injection location, and less radiation for the patient and the physician. The workflow of the 3D CartBox includes: MRI, segmentation, and EMM. Altogether this takes approximately 3 h, including the injections. Thereby it is a clinically feasible solution. Real-time integration of LGE-MRI during cardiac cell injection procedures could be a key to harnessing the full therapeutic effects of cardiac stem cell therapy. The 3D CartBox toolbox enables the use of all parameters (perfusion, fibrosis, myocardial wall thickening, myocardial tissue tagging) from a pre-procedural acquired MRI or other imaging modality (SPECT/CT) to guide the cell injection procedures. The 3D CartBox toolbox for image-guided cardiac cell injections has been awarded the BioMedical Materials Valorisation Grant, and will be made commercially available via the newly founded spin-off company CART-Tech: ‘Technical solutions to improve cardiac regenerative therapy’.

## Limitations

The use of the coronary ostia for landmark registration is potentially dangerous for the patient and therefore not suitable for clinical use of the toolbox, although clinical use of this method has been reported in the literature [21]. For clinical application of the toolbox in the future we aim to use other fiducial points. The use of three pigs in this study was found not to be sufficient to perform a quantitative analysis of the overlap between the infarct areas identified by EMM and LGE-MRI or perform a detailed analysis of the thresholds of the NOGA parameters in the areas with different infarct transmurality. A new study must be performed to explore these aspects by applying the 3D CartBox to a larger dataset. Although the criteria for good electromechanical mapping were adhered to during all procedures, acquiring more points might have resulted in a lower registration error. During the mapping procedure small respiratory-induced excursions of the catheter tip could be observed. Since the NOGA®XP system did not compensate for respiratory-induced motion of the catheter tip, this most likely affected the registration. Compensation for respiratory motion might be beneficial for future applications.

## Conclusion

We have developed the 3D CartBox toolbox for real-time registration of EMM and MRI data that combines the gold standard diagnostic imaging techniques and highly accurate cardiac navigation to guide cardiac cell injection procedures. The 3D CartBox toolbox shows promising results but more research is necessary to specify the optimal injection location in order to maximise the improvement of cardiac function by cardiac regenerative therapy.


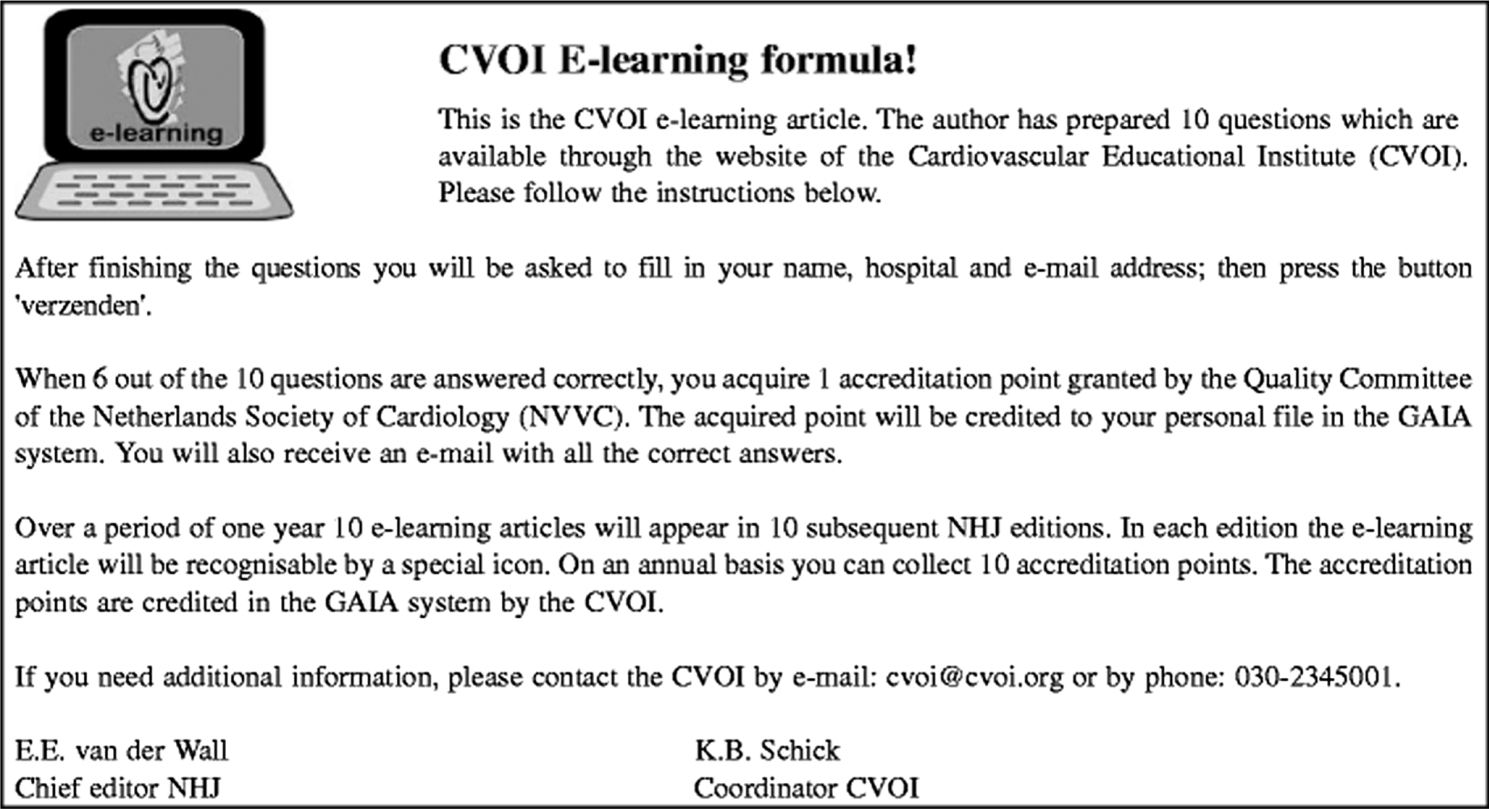


## Electronic supplementary material

Below is the link to the electronic supplementary material.

ESM 1 (DOCX 34.2 kb)

Fig. S1 The result of the area based infarct transmurality assessment by segment (A). The infarct transmurality projection on the cine mesh (B). The bottom figures (C and D) show 3D endocardial surface meshes with EMM points of an in-vivo dataset using the apex and left and right coronary ostia as landmarks for initial registration (C), and ICP registration (D). (GIF 157 kb)

High resolution image (TIFF 48313 kb)

Fig. S2 Cross-sectional view of the final registration results of an in vivo dataset. The green line represents the endocardial surface mesh and the yellow points are the EMM points. (GIF 365 kb)

High resolution image (TIFF 17776 kb)
