# Supplementary material for: Multimodality infarct identification for optimal image-guided intramyocardial cell injections
Source: Neth Heart J. 2014 Oct 21;22(11):493–500. doi: 10.1007/s12471-014-0604-2 (PMC4391177; doi:10.1007/s12471-014-0604-2)
Supplement: Supplementary file 1 — (DOCX 34.2 kb) [file 12471_2014_604_MOESM1_ESM.docx]

**Supplementary data**

In this supplementary data we describe the workflow and the algorithms that are used to perform image-guided cell injections using the 3D CartBox image registration toolbox. The subsequent steps of the 3D CartBox toolbox are discussed are illustrated in Fig. 1 of the main text. All the steps of the workflow were applied to *in vivo* datasets. For each step both the purpose and the algorithms used are discussed and illustrated with images.

**Data acquisition**

The *in vivo* MRI images were acquired using a 1.5 Tesla Philips Medical Systems Achieva scanner with a commercially available five-channel phased array cardiac coil. All scans were performed using ECG gating. The cine and LGE scans were done with 25 and 1 (end-diastolic) phases per cardiac cycle, respectively, at the same slice positions and with the same slice orientation. The cine scans were performed using balanced fast field echo with repetition time [TR]/echo time [ET] = 2.9 ms/1.45 ms. Flip angle = 55°, voxel size = 2.43 x 2.43 mm, field of view [FOV] = 35 x 35 cm, 144 x 144 matrix, 5 mm slice thickness, 30 phases/R to R interval. LGE scans were taken with [TR]/[ET] = 4.61 ms/1.41 ms, flip angle = 15°, voxel size = 1.36 x 1.36 mm, field of view [FOV] = 35 x 35 cm, 256 x 256 matrix, 5 mm slice thickness.

1. **Data pre-processing**

**Purpose:** To assess the infarct transmurality from the LGE scans and project infarct transmurality values on the endocardial surface mesh of the left ventricle in order to guide catheter injections based on infarct transmurality data. Assign an infarct transmurality border zone to use for cell injections.

**Method:** Segmentation of the left ventricle on the short-axis cine and LGE MRI data was done in the end-diastolic phase in approximately 20 slices located from apex to base. The segmentations were done automatically and checked on the long-axis images using the freely available software Segment version 1.9 R2507 (<http://segment.heiberg.se>) [23] available for Matlab (MATLAB 2012a, The MathWorks Inc., Natick, MA, 2012). Segmentations were done to create a 3D surface mesh (cine mesh) of the left ventricular endocardium for surface registration and projection of the acquired data. Subsequently the myocardial infarct was segmented on the LGE images using the area based semi-automatic segmentation [24]. If necessary both the left ventricle and the infarct segmentations were manually adjusted by an experienced radiologist. The results of the infarct segmentation process in one slice are illustrated in Fig. 3b in the main text. Area based infarct transmurality values are calculated in 80 circumferential segments of all slices using the bullseye function of segment. The results of the infarct transmurality assessment are illustrated in Fig. S1a. The infarct transmurality data were projected on the cine-derived endocardial surface mesh using the TriScatteredInterp function of Matlab. The cine-derived surface mesh with projected infarct transmurality data is illustrated in Fig. S1b. The infarct transmurality projected on the endocardial surface mesh is used to calculate the infarct border zone using an in house developed treatment planning algorithm. The infarct border zone is defined as a 1 cm wide area over the 50% infarct transmurality isoline. Furthermore the endocardial surface mesh is used for registration of the EMM points, image-guided injection procedures, and post-processing. Landmark locations used for initial registration are the coronary ostia and apex locations. These locations were selected manually in the end-diastolic frames of the cine images using customised Matlab software.

1. **Registration**

**Purpose:** To combine the EMM dataset and the MRI dataset in order to 1) guide the EMM catheter based on the transmurality values derived from LGE MRI data and 2) combine the EMM and the LGE dataset for comparison. The initial registration step is used for a coarse alignment of the EMM and cine MRI mesh. The iterative closest point (ICP) registration is used to fit the EMM points to the endocardial surface.

**Method:**

*Initial registration*

Two sets of three 3D points consisting of the two locations of the coronary ostia and the apex measured from cine MRI ${(p}_{MRI})$ and EMM ${(p}_{EMM})$ serve as registration landmarks, and are used as input for the initial registration algorithm. The order of the landmarks in both sets of points is the same. The initial registration algorithm is adapted from published algorithms [19,20] and consists of nine steps:

1: Calculate the centroid (c) of each point set: $p_{cMRI}=\frac{1}{3}\sum_{i=1}^{3} {p_{MRI}}_{i}$ $p_{cEMM}=\frac{1}{3}\sum_{i=1}^{3} {p_{EMM}}_{i}$

2: Translate both sets of points to the origin: $q_{MRI}={p_{MRI}}_{i}-p_{cMRI}$ $q_{EMM}={p_{EMM}}_{i}-p_{cEMM}$

3: Calculate the 3x3 matrix H: $H= \sum_{i=1}^{3} {q_{MRI}}_{i}\cdot{q_{EMM}^{t}}_{i}$

4: Calculate the singular value decomposition of H: $H=UDV^{T}$

5: Multiply the determinants of V and U: $X=det(V)\cdot det(U)$

6: Determine the S matrix dependent of X: $S=\left\{ \begin{aligned} I if X=1 \\ diag\left( 1,1,-1 \right) if X=-1 \end{aligned} \right.$

7: Determine the rotation matrix: $R=USV^{T}$

8: Determine the translation vector: $T= p_{EMM}-R\cdot p_{MRI}$

9: Apply $R$ and $T$ to the EMM dataset: ${allpoints}_{EMM}=\left( R\cdot a{llpoints}_{EMM} \right)+T$

For optimal registration of the apex the algorithm was adapted to apply 10 times higher weighing of the apex compared with the coronary ostia by adding points to the apex location. After applying the rotation matrix and translation vector to ${(p}_{EMM})$ and the complete set of all the EMM points ${(allpoints}_{EMM})$, the orientation and location of ${(p}_{EMM})$ and ${(allpoints}_{EMM})$is similar to the ${(p}_{MRI})$ point set. The result of the initial registration of an *in-vivo* EMM dataset is illustrated in Fig. S1c and can be used as input for the second registration step of the 3D CartBox toolbox.

*Iterative closest point registration*

# The second registration step incorporated in 3D CartBox is the ICP method [21, 22]. In this method the coarsely registered 3 x n point set of the cine MRI $\boldsymbol{(p}_{\boldsymbol{MRI}}\boldsymbol{)}$ and a 3 x m point set of the EMM $\boldsymbol{(p}_{\boldsymbol{EMM}}\boldsymbol{)}$ are used as input. The ICP algorithm consists of three steps that are iteratively executed.

# 1: For all points in the EMM dataset compute the closest point in the MRI dataset using the distance function d:

#

$$\mathbf{d(MRI,}\mathbf{p}_{\boldsymbol{EMM}_{\boldsymbol{i}}}\mathbf{)}\boldsymbol{=}\min_{\boldsymbol{j\in\{1,\ldots,n\}}} \left\| \mathbf{p}_{\boldsymbol{MRI}_{\boldsymbol{j}}}\boldsymbol{-}\mathbf{p}_{\boldsymbol{EMM}_{\boldsymbol{i}}} \right\|$$

Let C be the resulting set of m closest points for each of the points in the EMM set. To align the EMM to the MRI dataset a transformation $\left( T \right)$ consisting of both a rotation $\left( R \right)$ and a translation $\left( T \right)$ is necessary. For each point the transformation is: $T\left( p_{EMM} \right)=R\cdot p_{EMM}+T,$ and per set of closest points the resulting distance after the transformation can be calculated: $p_{{MRI}_{i}}-T\left( p_{E{MM}_{i}} \right)=p_{M{RI}_{i}}-\left( R\cdot p_{EMM_{i}}+T \right)$. To calculate the rotation and translation to minimise the registration error, an error function needs to be optimised:

2: Optimise error function: ${argmin}_{R,T} f\left( R,T \right)=\frac{1}{m}\sum_{i=1}^{m} \left\| p_{M{RI}_{i}}-\left( R\cdot p_{EMM_{i}}+T \right) \right\|^{2}$

3: Apply the translation to the EMM dataset: $p_{E{MM}_{new}}={R\cdot p}_{EMM}+T$

After the third step of the ICP process the first step of the ICP process is carried out again using the results of the third step. These three steps are iteratively executed until the criteria to finish the process are reached. In the 3D CartBox toolbox, the finalisation criteria are set to a maximum number of iterations (100) or a resulting sum squared error (SSE) below ${1\cdot10}^{-15}mm$ . Using ICP the registration is based upon the points in the ${(p}_{EMM})$ point set that are closest to the points in the ${(p}_{MRI})$ point set. The results of the ICP registration in an in-vivo dataset are shown in Fig. S1d, and cross-sectional views of the registration are shown in Fig. S2.

*Manual registration*

Because ICP does not take into account the anatomical correctness of the registration the results of the ICP algorithm need to be checked and approved by the user. This is incorporated into the 3D CartBox workflow. In this way the rotations and translations that are suggested by the ICP algorithm are controlled, to be correct and beneficial for the registration. To allow experienced physicians to optimise the registration based on anatomy or additional landmarks that are acquired during the EMM procedure, the 3D CartBox facilitates a manual manipulation of the registration. This can be done by adjusting the rotation and translation of ${(p}_{EMM})$ interactively with six degrees of freedom. These are rotation and translation in the sagittal, coronal, and transverse plane. Rotations were limited to 10, 20 and 20 degrees, respectively, to prevent excess rotations.

1. **Post-processing**

**Purpose:** Present data using clinical standard visualisation techniques. **Method:** Create bullseye plots.

**Error assessment**

The accuracy of the registration was expressed by the registration error being the mean ± standard deviation of the shortest distance from each EMM point to the cine mesh surface. To show the relevance of this measure a cross-sectional view of the registration is shown in Fig. S2. The registration error of the registration shown in Fig. S2 is 3.54 ± 1.6 and was based on 79 EMM points.
