# Supplementary figures and images for: Multimodality infarct identification for optimal image-guided intramyocardial cell injections
Source: Neth Heart J. 2014 Oct 21;22(11):493–500. doi: 10.1007/s12471-014-0604-2 (PMC4391177; doi:10.1007/s12471-014-0604-2)

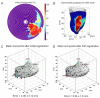

Supplement: Supplementary file 2 — The result of the area based infarct transmurality assessment by segment (A). The infarct transmurality projection on the cine mesh (B). The bottom figures (C and D) show 3D endocardial surface meshes with EMM points of an in-vivo dataset using the apex and left and right coronary ostia as landmarks for initial registration (C), and ICP registration (D). (GIF 157 kb) [file 12471_2014_604_Fig5_ESM.gif]

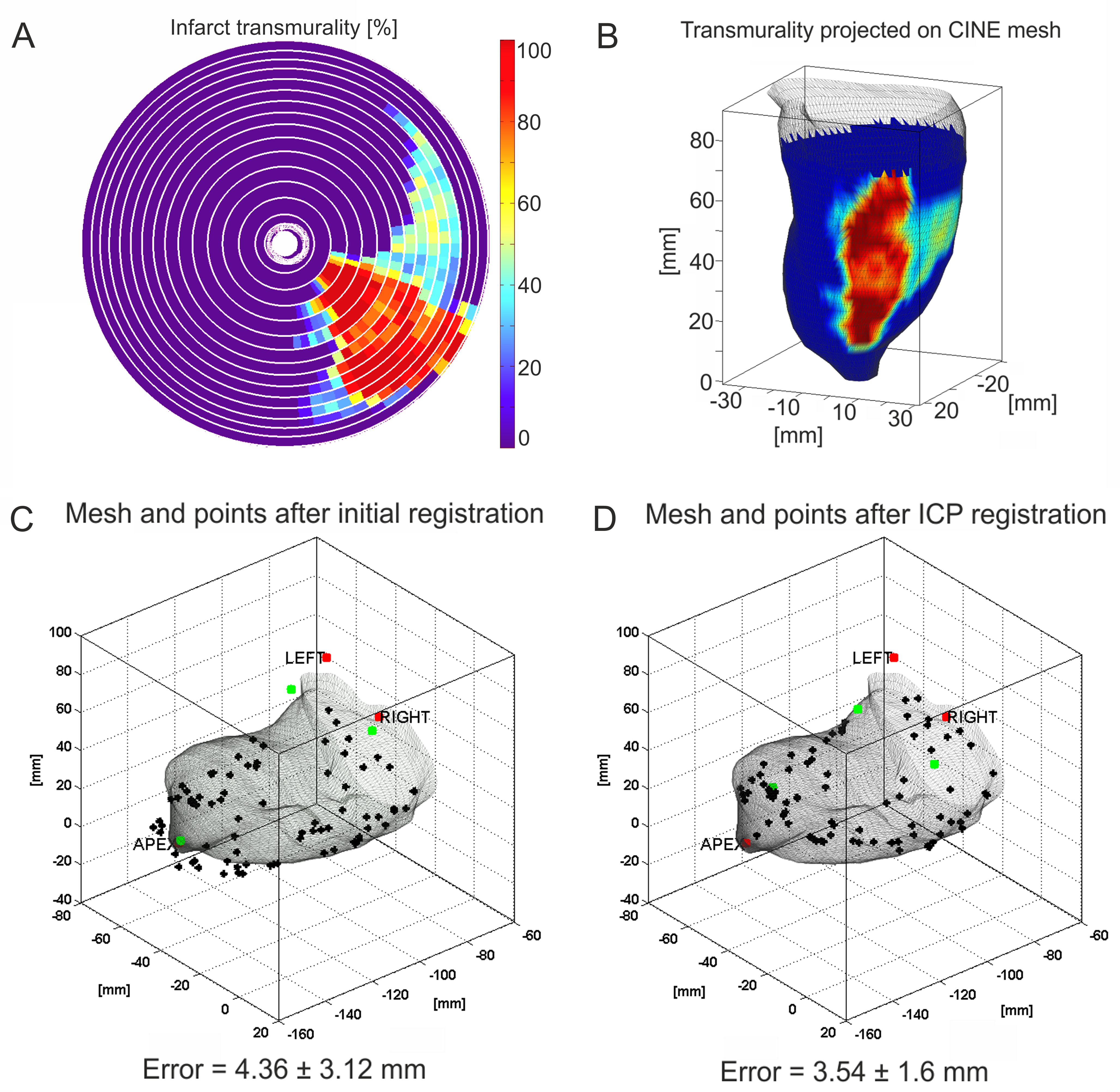

Supplement: Supplementary file 3 — High resolution image (TIFF 48313 kb) [file 12471_2014_604_MOESM2_ESM.tif]

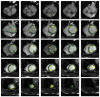

Supplement: Supplementary file 4 — Cross-sectional view of the final registration results of an in vivo dataset. The green line represents the endocardial surface mesh and the yellow points are the EMM points. (GIF 365 kb) [file 12471_2014_604_Fig6_ESM.gif]

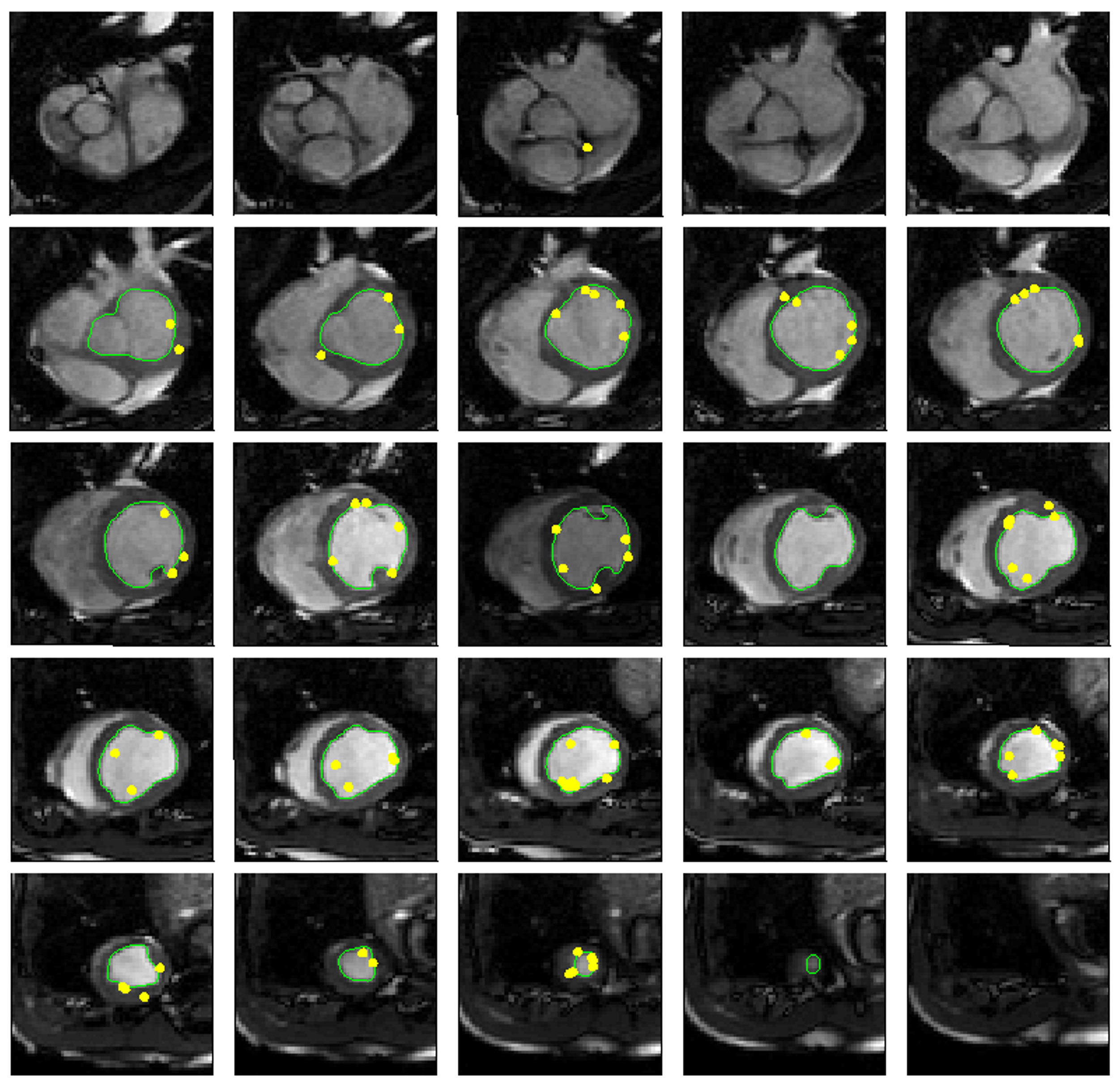

Supplement: Supplementary file 5 — High resolution image (TIFF 17776 kb) [file 12471_2014_604_MOESM3_ESM.tif]
